# Phosphatidylcholine Specific PLC-Induced Dysregulation of Gap Junctions, a Robust Cellular Response to Environmental Toxicants, and Prevention by Resveratrol in a Rat Liver Cell Model

**DOI:** 10.1371/journal.pone.0124454

**Published:** 2015-05-29

**Authors:** Iva Sovadinova, Pavel Babica, Hatice Böke, Esha Kumar, Andrew Wilke, Joon-Suk Park, James E. Trosko, Brad L. Upham

**Affiliations:** 1 Department of Pediatrics & Human Development; Center for Integrative Toxicology; and the Food Safety & Toxicology Center, Michigan State University, East Lansing, Michigan, 48824, United States of America; 2 Research Centre for Toxic Compounds in the Environment—RECETOX, Masaryk University, Kamenice 5, CZ62500, Brno, Czech Republic; 3 Department of Experimental Phycology and Ecotoxicology, Institute of Botany ASCR, Lidicka 25/27, CZ60200, Brno, Czech Republic; 4 Laboratory Animal Center, Daegu-Gyeongbuk Medical Innovation Foundation, Daegu, Korea; University of Colorado Denver, UNITED STATES

## Abstract

Dysregulation of gap junctional intercellular communication (GJIC) has been associated with different pathologies, including cancer; however, molecular mechanisms regulating GJIC are not fully understood. Mitogen Activated Protein Kinase (MAPK)-dependent mechanisms of GJIC-dysregulation have been well-established, however recent discoveries have implicated phosphatidylcholine-specific phospholipase C (PC-PLC) in the regulation of GJIC. What is not known is how prevalent these two signaling mechanisms are in toxicant/toxin-induced dysregulation of GJIC, and do toxicants/toxins work through either signaling mechanisms or both, or through alternative signaling mechanisms. Different chemical toxicants were used to assess whether they dysregulate GJIC *via* MEK or PC-PLC, or both Mek and PC-PLC, or through other signaling pathways, using a pluripotent rat liver epithelial oval-cell line, WB-F344. Epidermal growth factor, 12-O-tetradecanoylphorbol-13-acetate, thrombin receptor activating peptide-6 and lindane regulated GJIC through a MEK1/2-dependent mechanism that was independent of PC-PLC; whereas PAHs, DDT, PCB 153, dicumylperoxide and perfluorodecanoic acid inhibited GJIC through PC-PLC independent of Mek. Dysregulation of GJIC by perfluorooctanoic acid and R59022 required both MEK1/2 and PC-PLC; while benzoylperoxide, arachidonic acid, 18β-glycyrrhetinic acid, perfluorooctane sulfonic acid, 1-monolaurin, pentachlorophenol and alachlor required neither MEK1/2 nor PC-PLC. Resveratrol prevented dysregulation of GJIC by toxicants that acted either through MEK1/2 or PC-PLC. Except for alachlor, resveratrol did not prevent dysregulation of GJIC by toxicants that worked through PC-PLC-independent and MEK1/2-independent pathways, which indicated at least two other, yet unidentified, pathways that are involved in the regulation of GJIC. In conclusion: the dysregulation of GJIC is a contributing factor to the cancer process; however the underlying mechanisms by which gap junction channels are closed by toxicants vary. Thus, accurate assessments of risk posed by toxic agents, and the role of dietary phytochemicals play in preventing or reversing the effects of these agents must take into account the specific mechanisms involved in the cancer process.

## Introduction

Gap junctional intercellular communication (GJIC) represents a key regulatory mechanism for the maintenance of tissue homeostasis, regulation of cell growth, differentiation and death [1,2]. Gap junctional channels are formed between adjacent cells by proteins termed, connexins, and allow direct cell-to-cell flux of small (<1–1.5 kDa) hydrophilic molecules, such as metabolites, nutrients, ions or second messengers [3,4]. Chronic impairment of GJIC caused by oncogene activation, endogenous cell-death-induced compensatory release of growth factors or by exposure to tumorigenic xenobiotics is strongly linked to the promoting phase of cancer [5,6]. Conversely, tumor suppressor genes and chemopreventive agents are known to reverse the inhibitory effects of tumor promoters or oncogenes, and restore cell-cell communication [7,8].

A number of chemicals are known to rapidly dysregulate GJIC, including a model tumor promoter, 12-O-tetradecanoylphorbol-13-acetate (TPA), biological toxins, organic solvents, environmental pollutants, pesticides, pharmaceuticals, peroxides, metals and others [9]. Despite numerous studies reporting modulation of GJIC by chemicals and endogenous or exogenous ligands, the underlying intracellular mechanisms responsible for rapid inhibition of connexin- based cell-cell communication have not been fully elucidated.

Regulation of GJIC through the phosphorylation of connexins has been the most extensively studied mechanism of GJIC regulation. Connexin43, the most studied connexin in phosphorylation studies, was identified as a substrate for many kinases, including mitogen activated protein kinases (MAPKs), protein kinase A (PKA), protein kinase C (PKC), casein kinase 1, Src-kinase or Akt [10–12]. Activation of MEK1/2, which is a MAPK-kinase, is considered to be a mechanism by which TPA and epidermal growth factor (EGF) dysregulates GJIC [13,14].

Recently, phospholipase-dependent mechanisms have been reported in the control of connexin43-based GJIC. Toxicants, such as PCB153 or dicumylperoxide (diCuOOH) or 1-methylanthracene (1-MeA), dysregulated GJIC through a phosphatidylcholine-specific phospholipase C (PC-PLC) mechanism [15–17]. Phosphatidylinositol-specific phospholipase C (PI-PLC) does not play a role in either PCB153 or 1-MeA induced dysregulation of GJIC [15,17], while the involvement of PI-PLC was not determined for diCuOOH. Unlike PI-PLC, the function of PC-PLC in tumorigenesis has not been extensively studied, yet there are reports indicating that PC-PLC plays a very significant role in cancer [18]. Questions that arise are: What is the prevalence PC-PLC in toxicant-induced dysregulation of GJIC? Is PC-PLC involved in the dysregulation of GJIC by toxicants known to inhibit GJIC through Mek, or are Mek-dependent and PC-PLC-dependent inhibition of GJIC by toxicants unique mechanisms that are always independent of each other? Are these two mechanisms prevalent in toxicant-induced dysregulation of GJIC or do toxicants more commonly dysregulate GJIC through other, yet to be determined mechanisms?

**Fig 1:**
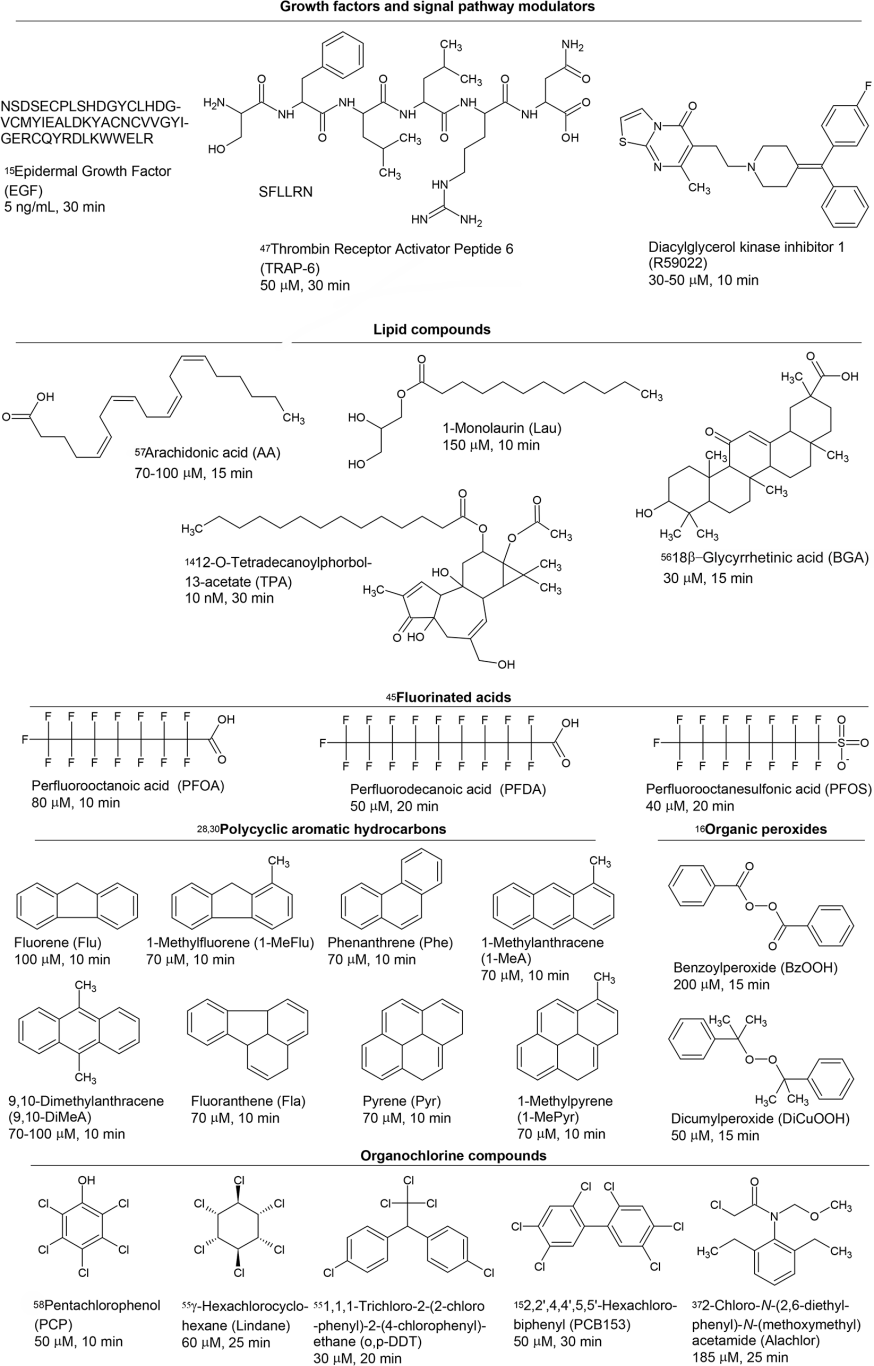
Structures, abbreviations, experimental doses and times of studied dysregulators of gap junctional intercellular communication. Superscript numbers of the following: [14–16,30,37,41,45,47,55–58] identify the references reporting a dysregulation of GJIC by these chemical in WB-F344 cells, with exception of Ref. 47, which used Rat-1 cells and Ref. 57, which used rat derived astrocytes.

In this report, we addressed these questions by determining if the dysregulation of connexin43-based GJIC in Fischer F344 rat liver epithelial cells (WB-F344), exposed to a selected set (25 compounds) of growth-regulating compounds, signal pathway modulators, environmental toxicants and potential tumor promoters (Fig 1), was mediated through either PC-PLC or MEK1/2, or both of these signaling proteins, or through other unidentified mechanisms. Several of these toxicants are known to dysregulate GJIC through either Mek or PC-PLC, but no study has yet to determine if these toxicants work through either one of both of these mechanisms, while the role of Mek and PC-PLC for many of the other GJIC-dysregulating toxicants are yet unknown.

The prevention of cancers through diet, and potentially by botanical supplements, is considered an important strategy in the control of this set of chronic diseases. Chemopreventive agents are known to prevent tumor promoters from dysregulating GJIC. Thus, another question that arises is: do anti-carcinogenic compounds prevent tumor promoters from dysregulating GJIC through Mek- and PC-PLC-dependent mechanisms or through general antioxidative mechanisms. For these experiments, we chose resveratrol, which is an antioxidant found in red wine and is associated with reduced rates of cardiovascular diseases and cancer [19]. Our results indicated that PC-PLC-dependent dysregulation of GJIC was a prevalent toxic mechanism and that there were at least two other mechanisms in addition to Mek and PC-PLC. Resveratrol prevented the dysregulation of GJIC by toxicants through Mek and PC-PLC but not through a third mechanism.

## Materials & Methods

### Chemicals

The chemicals used in this study were obtained from the following sources: resveratrol from CTMedChem (Bronx, NY); U0126 and D609 from Tocris Bioscience (Ellisville, MO); alachlor and lindane from Chem Service (West Chester, PA); arachidonic acid from Cayman Chemical (Ann Arbor, MI); 1-monolaurin (Lauricidin®) from Med-Chem Laboratories (Galena, IL); TPA from Biomol International (Plymouth Meeting, PA); 1-methylanthracene, 1-methylfluorene, benzoylperoxide (Luperox® A98), 18-β-glycyrrhetinic acid, dicumylperoxide, EGF, fluoranthene, fluorene, Lucifer Yellow CH dilithium salt(MW 457.25), pentachlorophenol, perfluorodecanoic acid (PFDA), perfluorooctane sulfonic acid (PFOSA), phenanthrene, pyrene and thrombin receptor activator peptide 6 (TRAP-6) from Sigma-Aldrich (St. Louis, MO); 9,10-dimethylanthracene, 1-methylpyrene, PFOA and 2,2',4,4',5,5'-hexachlorobiphenyl (PCB153) from Fluka (Buchs, Switzerland); 4,4'-(2,2,2-trichloroethane-1,1-diyl)bis(chlorobenzene) (DDT) from Supelco (Bellefonte, PA); R59022 from Calbiochem (La Jolla, CA); acetonitrile, dimethylsulfoxide (DMSO), ethanol and formaldehyde from Mallinckrodt Baker (Phillipsburg NJ).

### Cell line/Cell culture

The WB-F344 rat liver epithelial cell line was obtained from Drs. J. W. Grisham and M. S. Tsao of the University of North Carolina (Chapel Hill, NC) [20]. Cells were grown in D-medium formulated from commercially purchased modified Eagle's minimum essential medium (Formula No. 78–5470EF, Gibco Laboratories, Grand Island, NY) supplemented with 5% fetal bovine serum (Gibco Laboratories) and 10 μg/ml gentamicin (Gibco Laboratories). Cells were cultured on 35 mm diameter tissue culture plates (Corning Inc., Corning, NY) at 37°C in a humidified atmosphere containing 5% CO_2_ and 95% air. Bioassays were conducted with confluent cultures that were obtained after two to three days of growth.

This cell line derived from F344 rats was used for the following reasons. These WB-cells are diploid and non-tumorigenic [20] and have been extensively characterized for its expressed gap junction genes, namely connexin43, and functional GJIC using all available techniques in the absence and presence of well-known tumor promoters, growth factors, tumor suppressor genes and oncogenes to modulate GJIC [21]. WB-F344 cells are pluripotent, capable of differentiation into hepatocytes [22] and functional-contracting cardiomyocytes [23] and represent an ideal model for studying tumor promotion under the paradigm of stem-cell theory of cancer [24,25].

### Experimental design

Confluent WB-F344 cells grown on 35 mm dishes were first pretreated either with a PC-PLC inhibitor, D609 (50 μM, 20 min), a MEK1/2 inhibitor, U0126 (20 μM, 30 min), or resveratrol (100 μM, 15 min). After the pretreatment, the tested GJIC-dysregulator or vehicle was added to the dish for a specific incubation time and followed by a scalpel loading-dye transfer assay (note that the signal pathway inhibitor or resveratrol remained in the cell culture medium during the incubation period with the GJIC-dysregulator). The exposure regimes necessary to induce almost complete inhibition of GJIC (FOC<0.2–0.4) were determined in separate concentration- and time-response experiments (data not shown). The lowest concentration of GJIC-dysregulator and the shortest exposure time sufficient to significantly dysregulate GJIC were chosen and they are summarized in Fig 1. The tested chemicals at the applied doses were not cytotoxic after 1 h exposure as evaluated by Neutral Red uptake assay (data not shown), except the treatment with benzoylperoxide (200 μM) which induced about 50% decrease of Neutral Red uptake after 15 min exposure. The cytotoxic effects of benzoylperoxide are prevented by N-acetyl-L-(+)-cysteine (NAC) [16], therefore the experiments with benzoylperoxide were carried out with or without a 15 min pretreatment with 1 mM NAC prior to the addition of a toxicant to the cells.

To assure that the cells were responding to GJIC-dysregulators in a reproducible manner and that the signal-pathway inhibitors and resveratrol were able to induce significant effects, a system of positive controls (1-methylanthracene, D609 + 1-methylanthracene, resveratrol + 1-methylanthracene, EGF, and U0126 + EGF) was included in each experimental design besides the negative controls (vehicle, signal-pathway inhibitors or resveratrol without the GJIC-dysregulator). At least three independent experiments were conducted, each on a different day and cell population.

### Bioassay of GJIC

A scalpel loading-dye transfer (SL-DT) technique was adapted after the method of El-Fouly *et al*. [26]. After treatment with the chemicals, cells were washed with phosphate buffered saline containing 0.1 g/L calcium chloride and 0.1 g/L magnesium chloride (CaMgPBS) followed by the addition of 1 mg/ml of Lucifer-Yellow dissolved in CaMgPBS. The dye was introduced into the cells with three different lines of scalpel-based dye injection through the monolayer of confluent cells using a surgical steel scalpel blade. The transfer of dye through gap junctions was for three minutes, followed by a thorough rinse with CaMgPBS to remove extracellular dye, and then fixed with a 4% formalin solution in PBS. Migration of the dye in the cells was observed at 200X using a Nikon epifluorescence microscope equipped with a Nikon Cool Snap EZ CCD camera and the images digitally acquired using a Nikon NIS-Elements F2.2 imaging system. The fluorescence area of the dye migration from the scalpel line was quantified using ‘ImageJ’ image analysis program (National Institute of Health, Bethesda, MD). The data were reported as a fraction of the dye spread in the vehicle control (Fraction of the Control, FOC).

### Data and statistical analysis

The values were reported as an average ± S.D. from at least three independent SL-DT experiments. A one-way ANOVA and Dunnett’s post hoc test was computed using SigmaStat (SPSS Inc., Chicago, IL) to determine significance of differences (P<0.05) between a given dysregulator of GJIC and the following groups: (D609 + dysregulator), (U0126 + dysregulator), (resveratrol + dysregulator). The differences in the effects of D609, U0126 and resveratrol on the collection of all GJIC-dysregulators were further summarized in a principal component analysis (PCA) using Statistica for Windows 7.1 (StatSoft, Tulsa, OK, USA) and the results were presented in the component score plot of individual GJIC-dysregulators.

## Results

A mechanistic survey was conducted on 25 chemicals dysregulating GJIC (Fig 1) to determine if they interrupted gap junction function through MEK1/2, PC-PLC, and if the antioxidant, resveratrol, protected gap junction function from the GJIC-dysregulating effects of the tested toxicants. Structures of these toxicants are listed in Fig 1. GJIC was measured by SL-DT bioassay in a WB-F344 rat liver epithelial cells pre-incubated with an inhibitor of PC-PLC or MEK1/2, or resveratrol, followed by the addition of a GJIC-dysregulating chemical.

**Fig 2:**
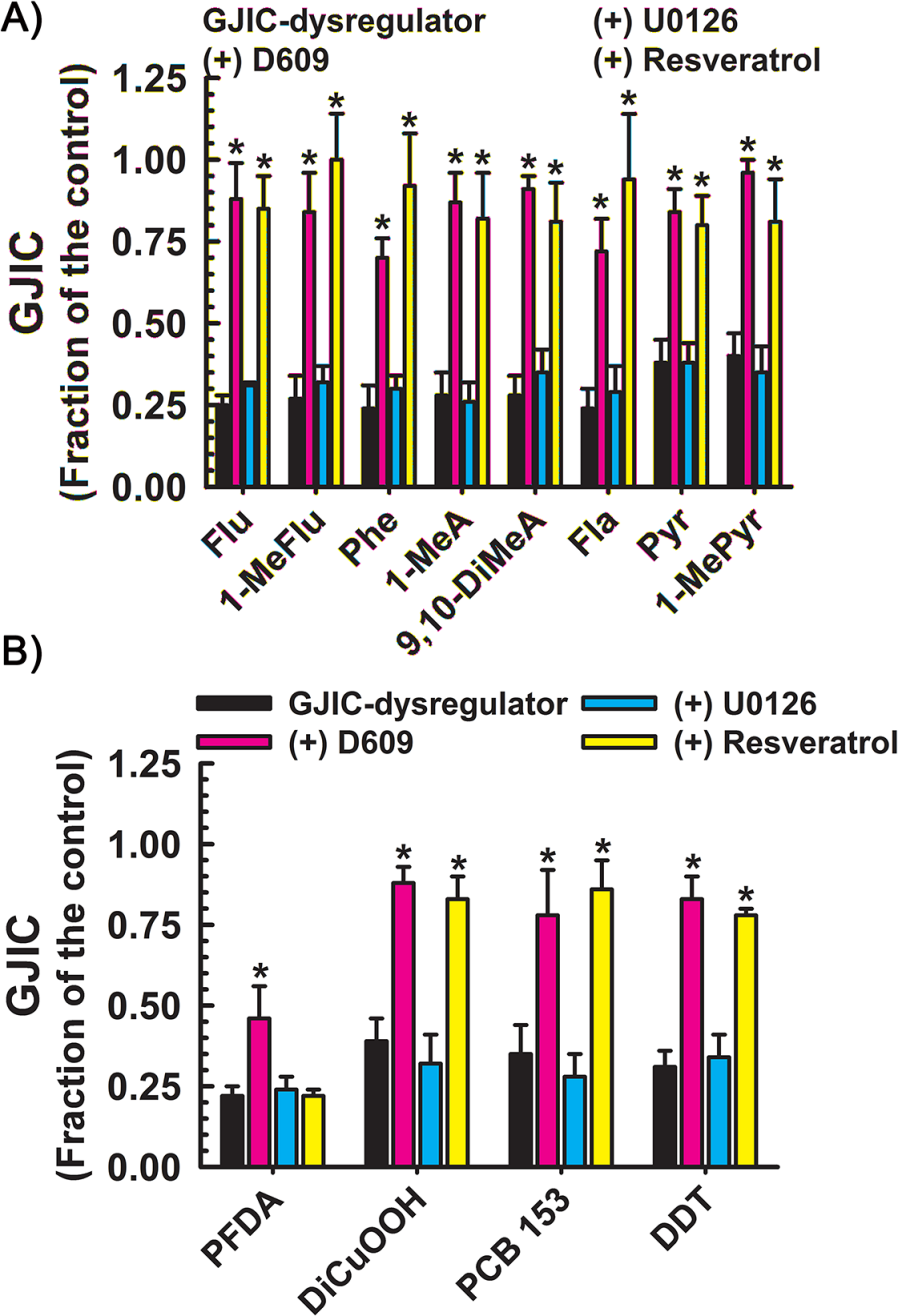
Dysregulation of GJIC through PC-PLC. The following compounds inhibited GJIC through PC-PLC: (**a)** Through the following PAHs: Flu (100 μM, 10 min), 1-MeFlu (70 μM, 10 min), Phe (70 μM, 10 min), 1-MeA (70 μM, 10 min), 9,10-DiMeA (100 μM, 10 min), Fla (70 μM, 10 min), Pyr (70 μM, 10 min) and 1-MePyr (70 μM, 10 min); (**b)** Other toxicants: PFDA (50 μM, 20 min), DiCuOOH (50 μM, 15 min), PCB 153 (50 μM, 30 min), and DDT (30 μM, 20 min). The cells were treated with inhibitors of PC-PLC (D609, 50 μM, 20 min) or MEK1/2 (U0126, 20 μM, 30 min), or resveratrol (100 μM, 15 min) before addition of GJIC-dysregulator. At least three independent experiments were averaged ± SD. An ANOVA was conducted for each GJIC-dysregulator followed by a Dunnett’s post-hoc test to determine significance (at P<0.05 as indicated by an *) from cells treated with only the GJIC-dysregulator. The F-values for Flu, 1-MeFlu, Phe, 1-MeA, 9,10-DiMeA, Fla, Pyr and 1-MeP were 71.8 (P<0.001), 75.6 (P<0.001), 57.7 (P<0.001), 737.3 (P<0.001), 74.2 (P<0.001), 58.4 (P<0.001), 67.4 (P<0.001) and 50.5 (P<0.001), respectively. The F-values for PFDA, DiCuOOH, PCB 153, and DDT were 13.1 (P = 0.002), 51.2 (P<0.001), 38.3 (P<0.001) and 87.5 (P<0.001), respectively.

**Fig 3:**
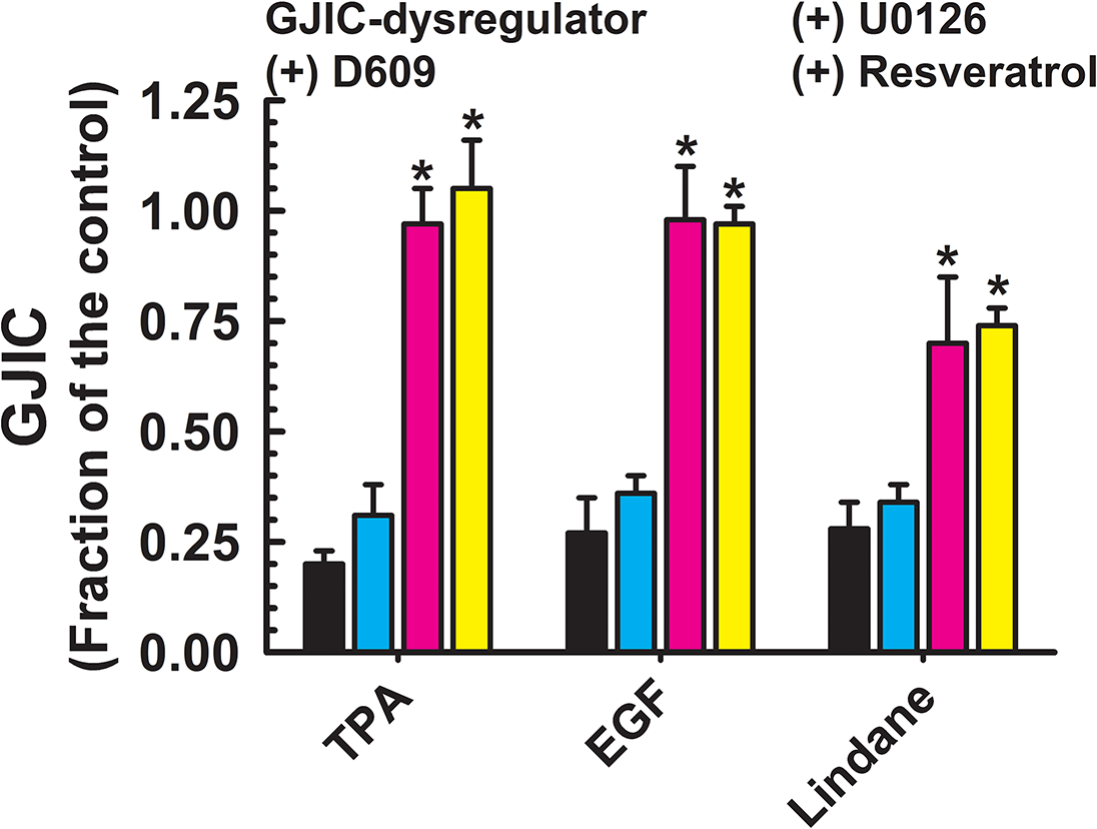
Dysregulation of GJIC through MEK1/2. The following compounds inhibited GJIC through MEK1/2: TPA (10 nM, 30 min), EGF (5 ng/ml, 30 min), TRAP-6 (50 μM, 30 min) and lindane (60 μM, 25 min). The cells were treated with inhibitors of MEK1/2 (U0126, 20 μM, 30 min) or PC-PLC (D609, 50 μM, 20 min), or resveratrol (100 μM, 15 min) before addition of GJIC-dysregulator. At least three independent experiments were averaged ± SD. An ANOVA was conducted for each GJIC-dysregulator followed by a Dunnett’s post-hoc test to determine significance (at P<0.05 as indicated by an *) from cells treated with only the GJIC-dysregulator. The F-values for TPA, EGF, TRAP-6 and lindane were 156.563 (P<0.001), 750.742 (P<0.001), 135.648 (P<0.001) and 36.717 (P<0.001), respectively.

**Fig 4:**
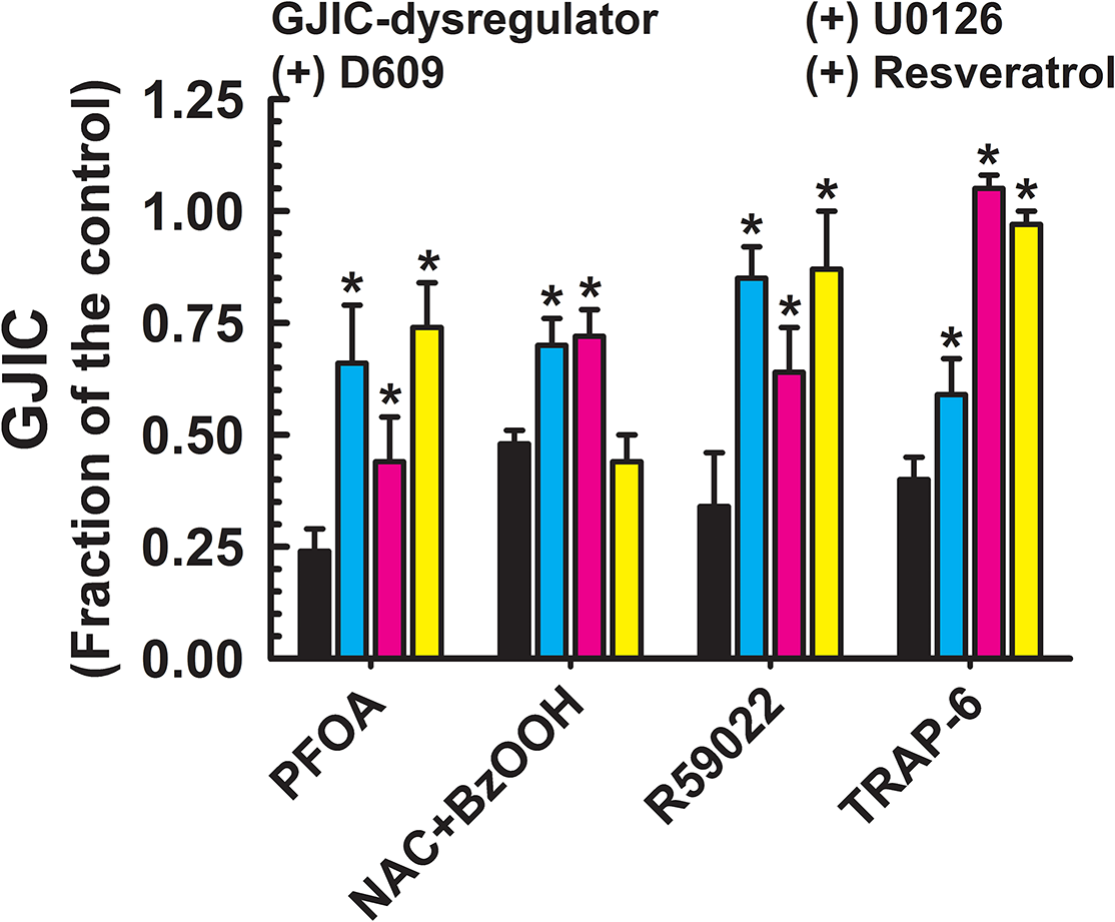
Dysregulation of GJIC through both MEK1/2 and PC-PLC. The following compounds inhibited GJIC through both MEK1/2 and PC-PLC: PFOA (80 μM, 10 min), NAC+BzOOH (cells were treated with 1 mM NAC for 15 min prior the addition of 200 μM BzOOH for 15 min), and R59022 (30–50 μM, 10 min). The cells were treated with inhibitors of PC-PLC (D609, 50 μM, 20 min) or MEK1/2 (U0126, 20 μM, 30 min), or resveratrol (100 μM, 15 min) before addition of GJIC-dysregulator. At least three independent experiments were averaged ± SD. An ANOVA was conducted for each GJIC-dysregulator followed by a Dunnett’s post-hoc test to determine significance (at P<0.05 as indicated by an *) from cells treated with only the GJIC-dysregulator. The F-values for PFOA and R59022 were 27.0 (P<0.001), 28.2 (P<0.001) and 20.9 (P<0.001), respectively.

**Fig 5:**
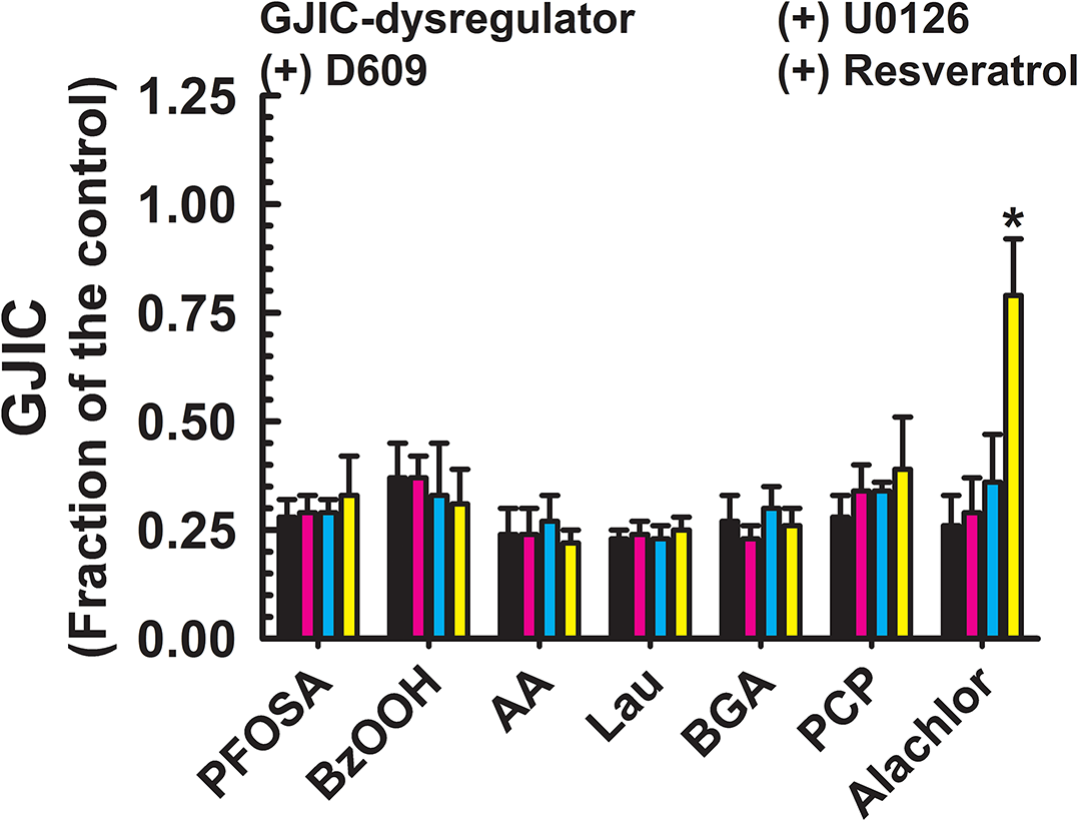
Dysregulation of GJIC through signaling pathways other than MEK1/2 or PC-PLC. The following compounds inhibited GJIC neither through MEK1/2 nor PC-PLC: PFOSA (40 μM, 20 min), BzOOH (200 μM, 15 min), AA (70–100 μM, 15 min), Lau (150 μM, 10 min), BGA (30 μμM, 15 min), PCP (50 μM, 10 min) and Alachlor (185 μM, 25 min). The cells were treated with inhibitors of PC-PLC (D609, 50 μM, 20 min) or MEK1/2 (U0126, 20 μM, 30 min), or resveratrol (100 μM, 15 min) before addition of GJIC-dysregulator. At least three independent experiments were averaged ± SD. An ANOVA was conducted for each GJIC-dysregulator followed by a Dunnett’s post-hoc test to determine significance (at P<0.05 as indicated by an *) from cells treated with only the GJIC-dysregulator. The F-values for PFOSA, BzOOH, AA, Lau, BGA, PCP and alachlor were 1.0 (P = 0.426), 0.6 (P = 0.628), 0.7 (P = 0.565), 0.6 (P = 0.617), 2.1 (P = 0.131), 1.9 (P = 0.162) and 58.6 (P<0.001), respectively.

All GJIC-dysregulators tested under the used exposure regimes induced statistically significant inhibition of GJIC with FOC values varying between 0.2–0.40 (Figs 2–5) when compared to the vehicle control (ANOVA, Dunnett’s post hoc test, P<0.05). The pretreatment of the cells with NAC resulted in a slight increase (about 11%) of GJIC in cells treated with benzoylperoxide (compare Figs 4 and 5). The levels of GJIC were measured in cells, first pretreated with either a signal pathway inhibitors or resveratrol, and then treated with a GJIC-dysregulator. These results were compared to the cells exposed only to the tested GJIC-dysregulator and significance of differences was evaluated by ANOVA and Dunnett’s post hoc test at P<0.05. Toxicants whose effects on GJIC were significantly attenuated by a PC-PLC inhibitor, D609, but not by MEK1/2 inhibitor, were fluorene, 1-methylfluorene, fluoranthene, phenanthrene, 1-methylanthracene, 9,10-dimethylanthracene, pyrene, 1-methylpyrene, PFDA, dicumylperoxide, PCB-153 and DDT (Fig 2A and 2B). With the exception of PFDA, D609 resulted in restoring GJIC to 0.70–0.96 FOC. D609 restored PFDA-induced dysregulation of GJIC only partially, by 24% (from 0.22 to 0.46 FOC).

Chemicals that were significantly prevented from GJIC-dysregulation by a MEK1/2 inhibitor, U0126, but not by a PC-PLC inhibitor, were TPA, EGF and lindane (Fig 3). The GJIC was restored to 0.70–0.98 FOC by pretreatment with U0126. The GJIC-dysregulating effects of PFOA, (NAC + benzoylperoxide), diacylglycerol kinase inhibitor I (R59022), and TRAP-6 were attenuated to a different extent (0.44–1.05 FOC) by both, PC-PLC and MEK1/2 inhibitors, D609 and U0126, respectively (Fig 4). Neither PC-PLC nor MEK1/2 inhibitor altered dysregulation of GJIC induced by PFOSA, BzOOH, arachidonic acid, 1-monolaurin, 18-β-glycyrrhetinic acid, pentachlorophenol, and alachlor (Fig 5), and the level of GJIC remained between 0.23–0.37 FOC.

Resveratrol effectively prevented the dysregulation of GJIC by all toxicants that worked through either a PC-PLC- (Fig 2A and 2B), MEK1/2- (Fig 3), or both PC-PLC and MEK1/2-dependent pathway (Fig 4), except for PFDA (Fig 2B) and benzoylperoxide in the cells pretreated with NAC (Fig 4). Resveratrol also prevented the dysregulation of GJIC by alachlor, but all other compounds that dysregulated GJIC through a PC-PLC-independent and MEK1/2-independent mechanism were unaffected by resveratrol (Fig 5). Resveratrol alone had no affect on GJIC nor did it reverse the inhibitory effects of GJIC if added after the addition of the dysregulator (data not shown).

**Fig 6:**
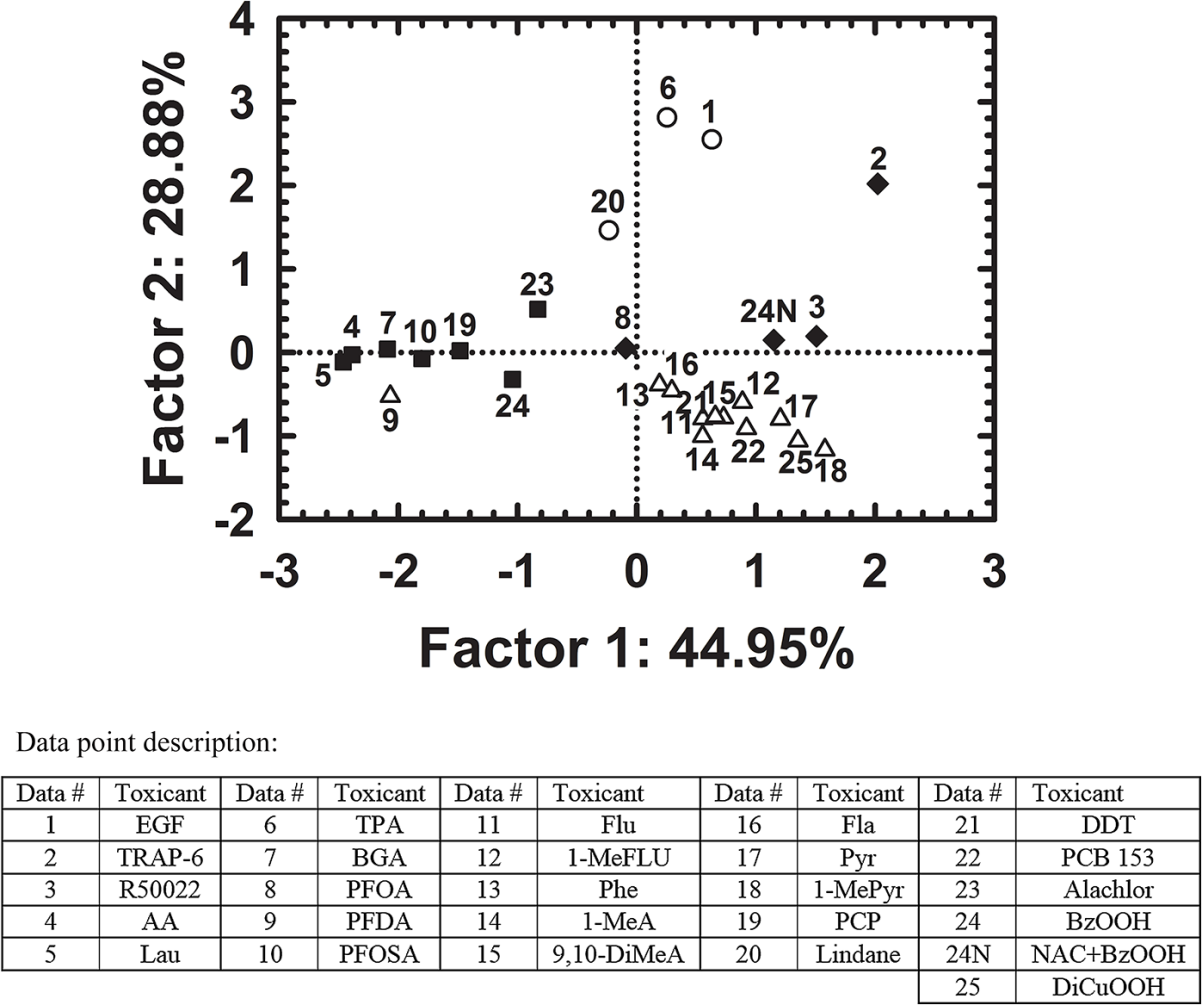
Component score plot from principal component analysis showing distribution of different GJIC-dysregulators based on their effects on GJIC and alteration of these effects by D609, U0126 or resveratrol. Symbols represent different groups of GJIC-dysregulators: (**triangles** = PC-PLC-dependent compound, (**circles** = MEK1/2-dependent compounds), (**diamonds** = both PC-PLC- and MEK1/2-dependent compounds), (**squares** = neither PC-PLC- nor MEK1/2-dependent compounds).

A principal component analysis (PCA) was conducted on all data using four variables of FOC values from experiments with (1) dysregulator, (2) D609 + dysregulator, (3) U0126 + dysregulator or (4) resveratrol + dysregulator). The first two principal components in the component score plot of individual dysregulators of GJIC explained 74% of the variance (Fig 6). The four different symbols represent GJIC-dysregulators as grouped in the Figs 1–4 based on the effects of D609 and U0126 (PC-PLC-dependent chemicals, MEK1/2- dependent chemicals, both PC-PLC and MEK1/2-dependent chemicals, PC-PLC- and MEK1/2-independent chemicals). The PCA plot indicated a cluster of all PC-PLC-dependent GJIC-dysregulators except PFDA, and also for PC-PLC- and MEK1/2-independent GJIC-dysregulators except for alachlor. MEK1/2-dependent GJIC-dysregulators, TPA and EGF clustered together, but another MEK1/2-dependent chemical, lindane, showed to be an outlier from this cluster. PC-PLC and MEK1/2-dependent chemicals were both scattered in the plot, with PFOA and R59022 closer to the PC-PLC-dependent group, but TRAP-6 was in proximity to the MEK1/2 dependent group.

## Discussion

Intercellular communication *via* opened-gap junction channels is critical in maintaining the homeostasis of a tissue. Although transient closure of gap junctions in response to a growth factor or cytokine is normal, chronic closure of the channels by persistent exposure to xenobiotics, cytokines and growth factors interrupts tissue homeostasis and is characteristic of the hyperproliferative states of growth and development diseases, such as cancer [6,9,27]. The mechanisms involved in the regulation of gap junction channels are not fully understood, but MEK1/2 and PC-PLC are key signal transduction proteins known to be involved. The role of PC-PLC in the regulation of GJIC has only been recently determined [15–17] for a very limited number of compounds, namely 1-methylanthracene, dicumylperoxide and PCB-153. Thus, the prominence of PC-PLC in toxicant-induced inhibition of GJIC is not known. Our results indicated that the dysregulation of GJIC through a PC-PLC-dependent mechanism is a robust response of an oval-like rat liver epithelial cell line to many toxicants. Of the 25 compounds tested, 15 of them likely required PC-PLC in the dysregulation of GJIC, and three of these compounds also required MEK1/2.

Eight of these 25 compounds were low molecular weight polycyclic aromatic hydrocarbons (PAHs). Although the lower molecular weight PAHs are the most prominent PAHs found in the environment, these PAHs, which have two to four aromatic rings, have been largely ignored by the research community in assessing adverse health effects due to their lack of genotoxicity [8,17,27]. However, lower molecular weight-PAHs are biologically active and known to induce signaling pathways that control gene expression. Thus, these low molecular weight PAH-compounds must be considered as “epigenetic toxicants” [27]. In particular, low molecular weight-PAHs were shown to be effective inhibitors of GJIC, and the GJIC-inhibitory properties were strongly linked to the existence of a bay or bay-like regions on the PAH [27–30], which also correlated with the induction of arachidonic acid release and activation of MAPKs [17,31]. Mixtures of PAHs exhibit an additive effect on the inhibition of GJIC suggesting a common mechanism of action [32]. The PAHs tested in this study (9,10-dimethylanthracene, fluorene, 1-methylfluorene, fluoranthene, phenanthrene, pyrene, 1-methylpyrene) all inhibited GJIC through a resveratrol sensitive, PC-PLC-dependent mechanism that was independent of MEK1/2 activation, which is consistent with a common-mechanism hypothesis.

Dysregulation of GJIC through a PC-PLC-dependent mechanism was not unique to the PAHs, but also to several other very prominent environmental toxicants (PCB153 and PFDA), pesticide (DDT) and the oxidant (dicumylperoxide). Similar to lower molecular weight PAHs, the effects of these toxicants on GJIC were also prevented by pretreatment with resveratrol. With the exception of PFDA, inhibition of PC-PLC by D609 almost completely prevented toxicant-induced dysregulation of GJIC (0.78–0.88 FOC), but PFDA-induced dysregulation of GJIC was only partially prevented by D609 (0.46 FOC). Correspondingly, the PCA analysis placed PFDA out of the cluster of PC-PLC-dependent chemicals and close to the cluster of toxicants that were dependent on neither PC-PLC- nor MEK1/2.

Ligands of the receptor tyrosine kinase-Raf-pathway, such as EGF, or PKC-activators, such as TPA, result in MEK1/2-dependent inhibition of GJIC [14,33,34], and is the most studied mechanism of GJIC-dysregulation. EGF and TPA also induce the phosphorylation of connexin43 [10,13,35,36]. Our results confirm published data indicating that EGF and TPA dysregulate GJIC through MEK1/2, but our current results indicated that PC-PLC was not involved. Although lindane is known to dysregulate GJIC [37], this is the first time implicating MEK1/2 as the underlying mechanism of lindane inhibition of GJIC. The importance of these findings is that these compounds dysregulated GJIC specifically through MEK1/2 and not through PC-PLC. Thus, PC-PLC and MEK1/2 regulation of GJIC are two separate intracellular signaling mechanisms regulating GJIC.

Although MEK1/2 appears to be a GJIC-regulatory pathway, the activation of MEK1/2 alone is not necessarily sufficient for the dysregulation of GJIC [38]. Most of the chemicals investigated in this study are also reported to activate the MAPK/ERK pathway in different cell types, including WB-F344 cells: TRAP-6 [39], arachidonic acid [40], PFOA, [41], 1-methylanthracene [17,31], pentachlorophenol [42], lindane [43], DDT [44], PCB153 [15], benzoylperoxide and dicumylperoxide [16]; yet, not all of these compounds dysregulate GJIC through a MEK1/2-ERK-dependent pathway (See Figs 2 and 5). As noted above, connexin43 has known phosphorylation sites specific to MAPKs, but the activation of MEK1/2, ERK1/2 and p38 by 1-MeA did not alter the phosphorylation status of Connexin43 as detected by Western blots [17,31]. Collectively, these results indicate that the dysregulation of GJIC by MEK1/2-dependent pathway requires another essential cell signaling protein.

Two compounds, PFOA and the diacylglycerol kinase inhibitor I, R59022, inhibited GJIC that depended, in part on both PC-PLC and MEK1/2. PFOA has been shown to activate MAPK/ERK [41] and inhibit GJIC [45]. Additionally, the inhibition of diacylglycerol kinase by R59022 results in an accumulation of diacylglycerol and activation of PKC [46], and we may speculate that possible PKC-dependent activation of MAPK/ERK pathway could explain the effects of MEK1/2 inhibitor on GJIC-dysregulation induced by R59022. However, these two chemicals were also PC-PLC- and resveratrol-dependent, which indicates that these toxicants can dysregulate gap junction function through multiple pathways. TRAP-6 clearly dysregulated GJIC through a MEK1/2 mechanism, but was partially dependent on PC-PLC-inhibitor as indicated by a 19% increase in GJIC by pretreatment with D609, however this small D609 effect did not remove this toxicant from the cluster of MEK-dependent compounds in the PCA plot. Interestingly, TRAP-6 was previously reported to dysregulate connexin43-mediated GJIC in Rat-1 fibroblasts through a PI-PLC-induced decrease of membrane phosphatidylinositol 4,5-bisphosphate, a mechanism most likely not-involving MAPK pathway [47].

Another significant result was that 7 of the 25 compounds tested inhibited GJIC through mechanisms independent of either PC-PLC or MEK1/2. These compounds included PFOSA, benzoylperoxide, arachidonic acid, 1-monolaurin, 18β-glycyrrhetinic acid, pentachlorophenol, and alachlor. Except for alachlor, resveratrol did not prevent the dysregulation of GJIC. These results clearly indicate at least two other mechanism are involved in the closure of gap junction channels in that one of these mechanisms was independent of MEK1/2, PC-PLC and resveratrol, and the other dependent on resveratrol. Possible candidates might include PKA, PKC, Akt or p38 [10–12,48], phospholipases [47] and redox-dependent regulatory mechanisms [49].

The identification of underlying mechanisms regulating GJIC is important in determining more accurate assessments on adverse health effects of environmental toxicants, as well as developing potential preventive health strategies. Ingestion of natural compounds found in dietary food or supplements is one major approach in developing more effective prevention strategies to the adverse health effects of exposures to environmental toxicants. Due to numerous reports in the prevention of cardiovascular diseases, cancer, and the aging processes in several organisms, resveratrol is one compound that has received considerable attention in the research field [19]. Several reports have demonstrated resveratrol can modulate toxic effects of epigenetic compounds on GJIC [50,51]. Our results indicated that resveratrol prevented compounds from inhibiting GJIC through PC-PLC and MEK1/2. Possibly these two pathways are redox sensitive, which are specific to resveratrol. NAC, also an antioxidant, did not prevent BzOOH-induced dysregulation of GJIC [16]. NAC also did not prevent 1-MeA-induced dysregulation of GJIC (data not shown). Although we used higher concentrations of resveratrol for our mechanistic studies, we previously showed that *in vitro* concentrations of resveratrol as low as 25 μM significantly prevented inhibition of GJIC by dicumylperoxide indicating physiologic significance [16], particularly if other food- or supplement-based antioxidative compounds can exhibit similar activities. These results suggest that populations exposed to toxicants that dysregulate GJIC through PC-PLC- and MEK-dependent mechanisms could potentially benefit from dietary intake of resveratrol and similar compounds in the prevention of diseases that depend on the dysregulation of GJIC.

## Conclusions

**Fig 7:**
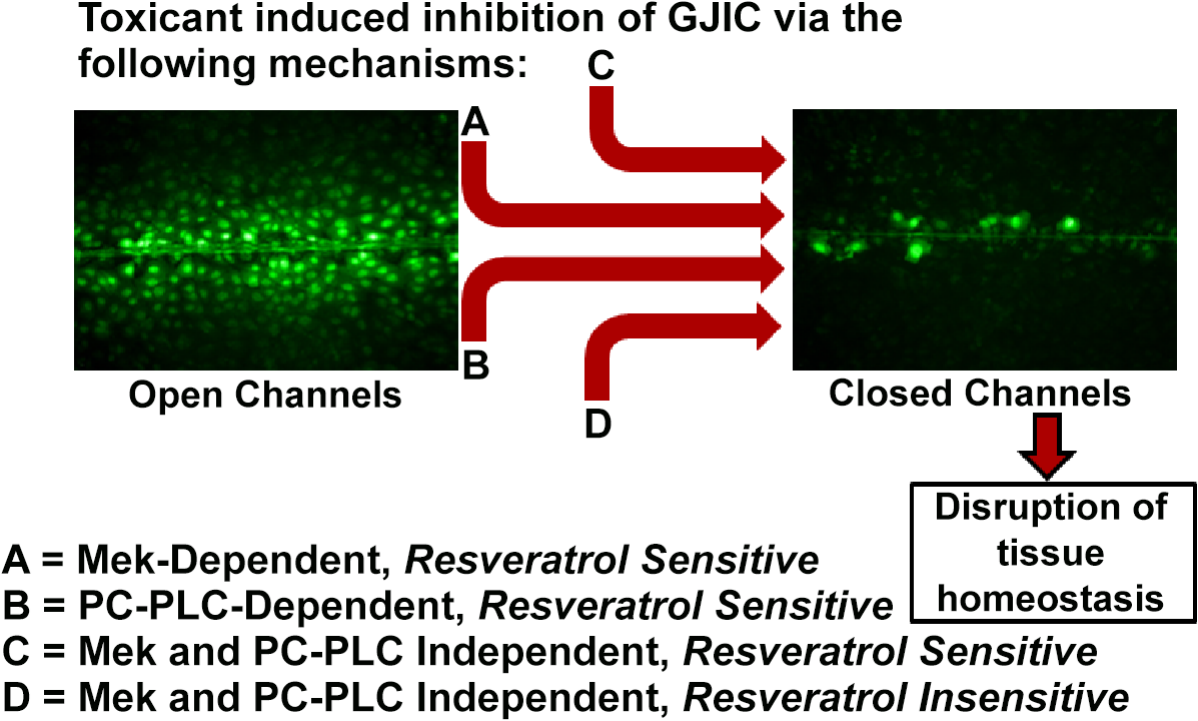
Summary of toxicant-dependent regulatory pathways of GJIC. Each of the four pathways are designated as **A.** Mek-dependent and resveratrol sensitive, **B.** Phosphatidylcholine-specific phospholipase C (PC-PLC) and resveratrol sensitive, **C.** Mek and PC-PLC independent and resveratrol sensitive, and **D.** Mek and PC-PLC independent and resveratrol insensitive.

The disruption of tissue homeostasis by compounds through the dysregulation of intercellular communication through gap junctions poses potential health risks. The dysregulation of GJIC occurs through multiple pathways (Fig 7), and the identification of these pathways within tissue types will be useful in the assessment of risks and benefits of toxicants and chemopreventive compounds. Considering that the liver is a major target for environmental toxicants, the use of the rat liver oval cell type is a good in vitro model system to begin an assessment of the underlying mechanisms involved in regulating GJIC. These cells are bipotent in hepatic tissue, capable of differentiation into hepatocytes and biliary duct cells [22], and represent an ideal *in vitro* model for studying tumor promotion in liver under the paradigm of stem-cell theory of cancer [24,25]. Our results indicate at least four different pathways are regulating GJIC (Fig 7), two of which depend on either PC-PLC or MEK1/2. The recently discovered PC-PLC-dependent mechanism was a robust and prevalent response of epithelial cells to environmental toxicants. Of the 25 compounds tested, 15 of them required PC-PLC in the dysregulation of GJIC, while three of these compounds also required MEK1/2. The function of PC-PLC in tumorigenesis has not been extensively studied, yet, there are reports indicating that PC-PLC plays a very significant role in cancer [18,52]. PC-PLC has also been specifically linked to the regulation of cellular differentiation [53] and apoptosis [54], which are processes strongly associated with tumor promotion. Furthermore, resveratrol effectively prevented the dysregulation of GJIC that worked through either MEK1/2 or PC-PLC indicating the potential to prevent an adverse effect of a toxicant through compounds that can be attained through diet. However, preventive effects by such compounds are apparently not universal considering that not all GJIC-regulatory mechanisms are equally affected by a chemopreventive agent, such as resveratrol (Fig 7). These results, which are relevant to liver tissue, also demonstrate an important conceptual way of integrating extra- intra- and gap junctional inter-cellular communication mechanisms to maintain tissue homeostasis.

## Supporting Information

S1 Dataset (XLSX)
