# Correction: Phosphatidylcholine Specific PLC-Induced Dysregulation of Gap Junctions, a Robust Cellular Response to Environmental Toxicants, and Prevention by Resveratrol in a Rat Liver Cell Model

**DOI:** 10.1371/journal.pone.0137599

**Published:** 2015-09-01

**Authors:** Faiz Ahmed Raza, Shafiq ur Rehman, Ruqyya Khalid, Jameel Ahmad, Sajjad Ashraf, Mazhar Iqbal, Shahida Hasnain

Figs 2, 3, 4, and 5 are each missing an internal color legend. The authors have provided a corrected version of each figure here.

**Fig 2:**
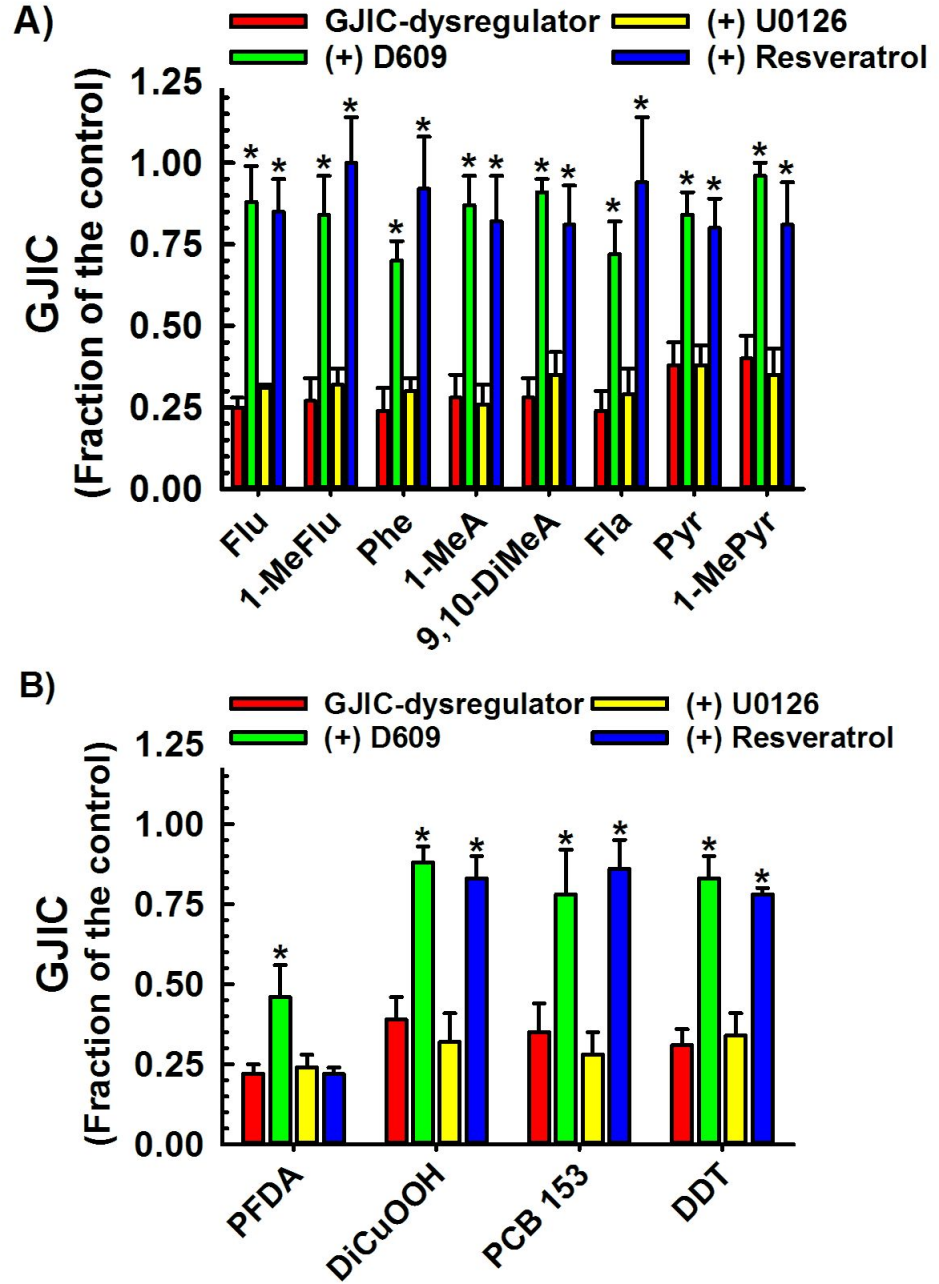
Dysregulation of GJIC through PC-PLC. The following compounds inhibited GJIC through PC-PLC: (**a)** Through the following PAHs: Flu (100 μM, 10 min), 1-MeFlu (70 μM, 10 min), Phe (70 μM, 10 min), 1-MeA (70 μM, 10 min), 9,10-DiMeA (100 μM, 10 min), Fla (70 μM, 10 min), Pyr (70 μM, 10 min) and 1-MePyr (70 μM, 10 min); (**b)** Other toxicants: PFDA (50 μM, 20 min), DiCuOOH (50 μM, 15 min), PCB 153 (50 μM, 30 min), and DDT (30 μM, 20 min). The cells were treated with inhibitors of PC-PLC (D609, 50 μM, 20 min) or MEK1/2 (U0126, 20 μM, 30 min), or resveratrol (100 μM, 15 min) before addition of GJIC-dysregulator. At least three independent experiments were averaged ± SD. An ANOVA was conducted for each GJIC-dysregulator followed by a Dunnett’s post-hoc test to determine significance (at P<0.05 as indicated by an *) from cells treated with only the GJIC-dysregulator. The F-values for Flu, 1-MeFlu, Phe, 1-MeA, 9,10-DiMeA, Fla, Pyr and 1-MeP were 71.8 (P<0.001), 75.6 (P<0.001), 57.7 (P<0.001), 737.3 (P<0.001), 74.2 (P<0.001), 58.4 (P<0.001), 67.4 (P<0.001) and 50.5 (P<0.001), respectively. The F-values for PFDA, DiCuOOH, PCB 153, and DDT were 13.1 (P = 0.002), 51.2 (P<0.001), 38.3 (P<0.001) and 87.5 (P<0.001), respectively.

**Fig 3:**
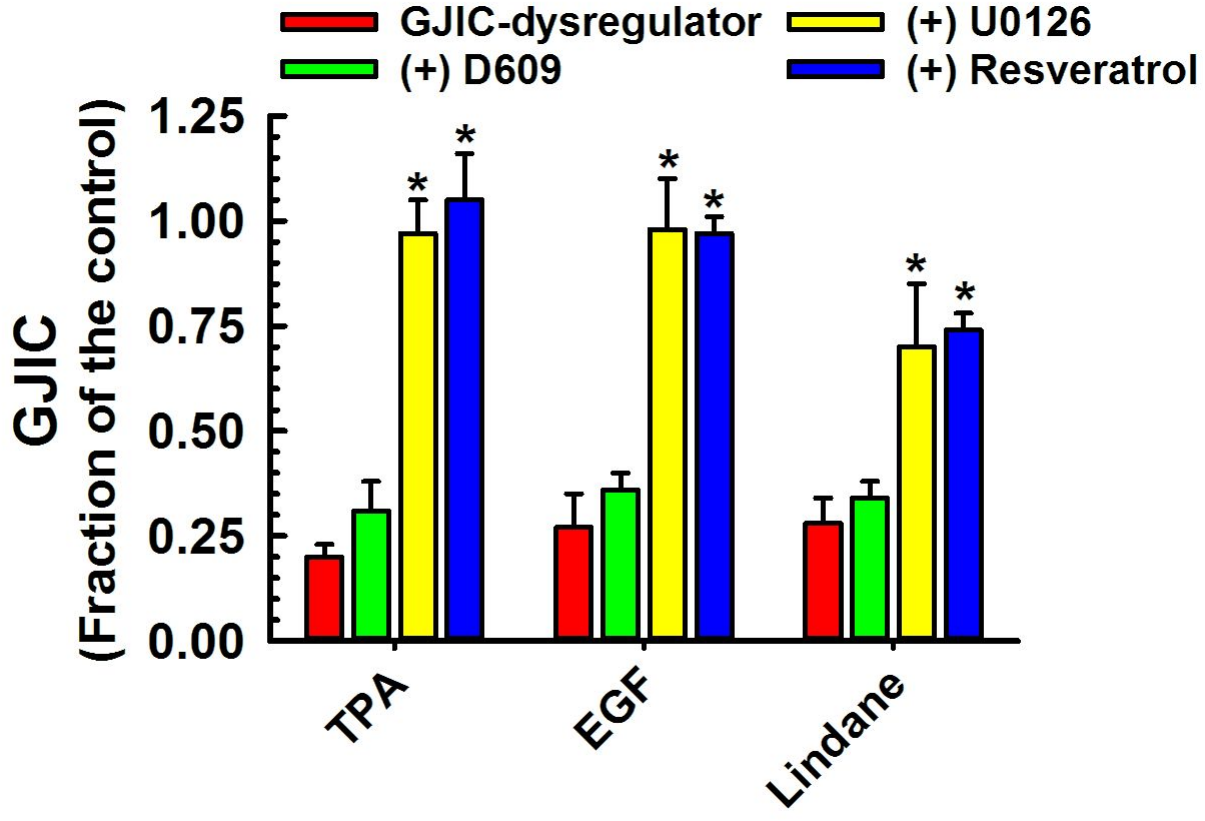
Dysregulation of GJIC through MEK1/2. The following compounds inhibited GJIC through MEK1/2: TPA (10 nM, 30 min), EGF (5 ng/ml, 30 min), TRAP-6 (50 μM, 30 min) and lindane (60 μM, 25 min). The cells were treated with inhibitors of MEK1/2 (U0126, 20 μM, 30 min) or PC-PLC (D609, 50 μM, 20 min), or resveratrol (100 μM, 15 min) before addition of GJIC-dysregulator. At least three independent experiments were averaged ± SD. An ANOVA was conducted for each GJIC-dysregulator followed by a Dunnett’s post-hoc test to determine significance (at P<0.05 as indicated by an *) from cells treated with only the GJIC-dysregulator. The F-values for TPA, EGF, TRAP-6 and lindane were 156.563 (P<0.001), 750.742 (P<0.001), 135.648 (P<0.001) and 36.717 (P<0.001), respectively.

**Fig 4:**
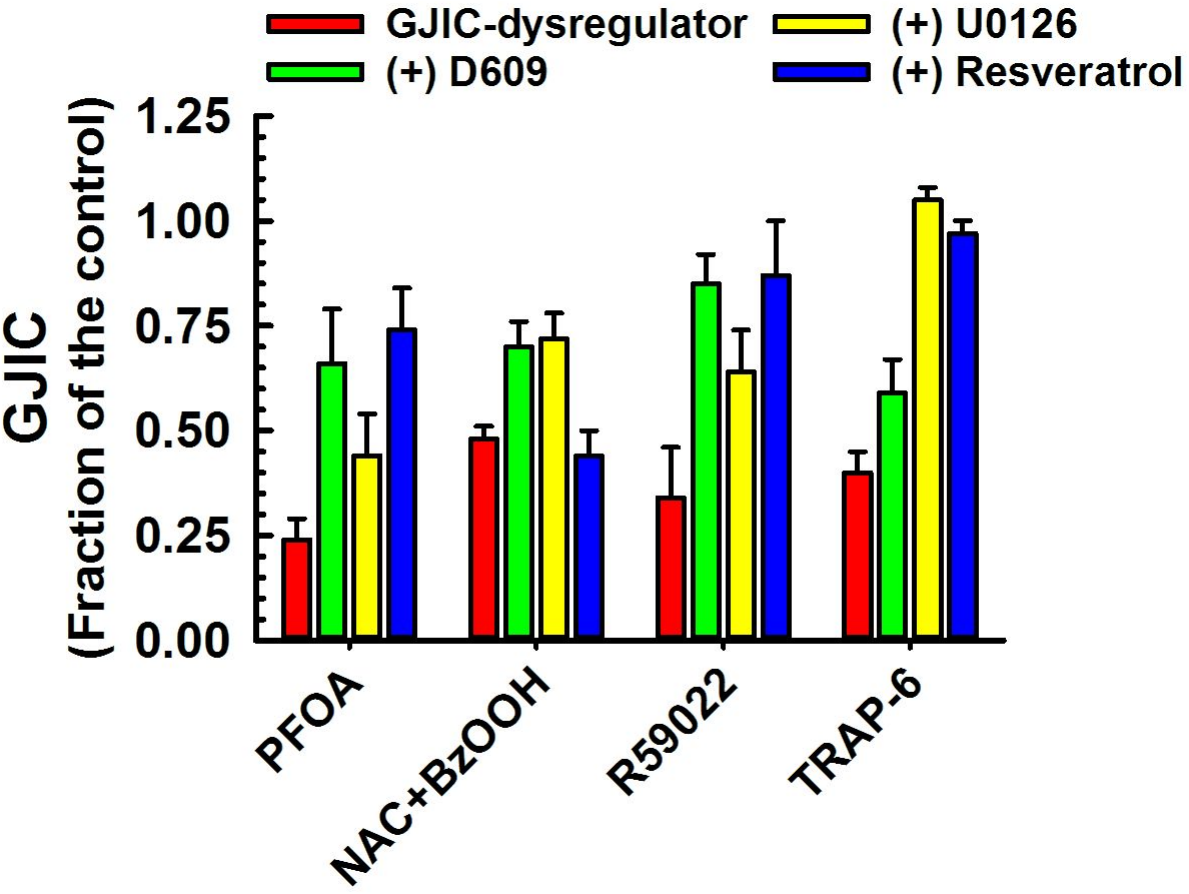
Dysregulation of GJIC through both MEK1/2 and PC-PLC. The following compounds inhibited GJIC through both MEK1/2 and PC-PLC: PFOA (80 μM, 10 min), NAC+BzOOH (cells were treated with 1 mM NAC for 15 min prior the addition of 200 μM BzOOH for 15 min), and R59022 (30–50 μM, 10 min). The cells were treated with inhibitors of PC-PLC (D609, 50 μM, 20 min) or MEK1/2 (U0126, 20 μM, 30 min), or resveratrol (100 μM, 15 min) before addition of GJIC-dysregulator. At least three independent experiments were averaged ± SD. An ANOVA was conducted for each GJIC-dysregulator followed by a Dunnett’s post-hoc test to determine significance (at P<0.05 as indicated by an *) from cells treated with only the GJIC-dysregulator. The F-values for PFOA and R59022 were 27.0 (P<0.001), 28.2 (P<0.001) and 20.9 (P<0.001), respectively.

**Fig 5:**
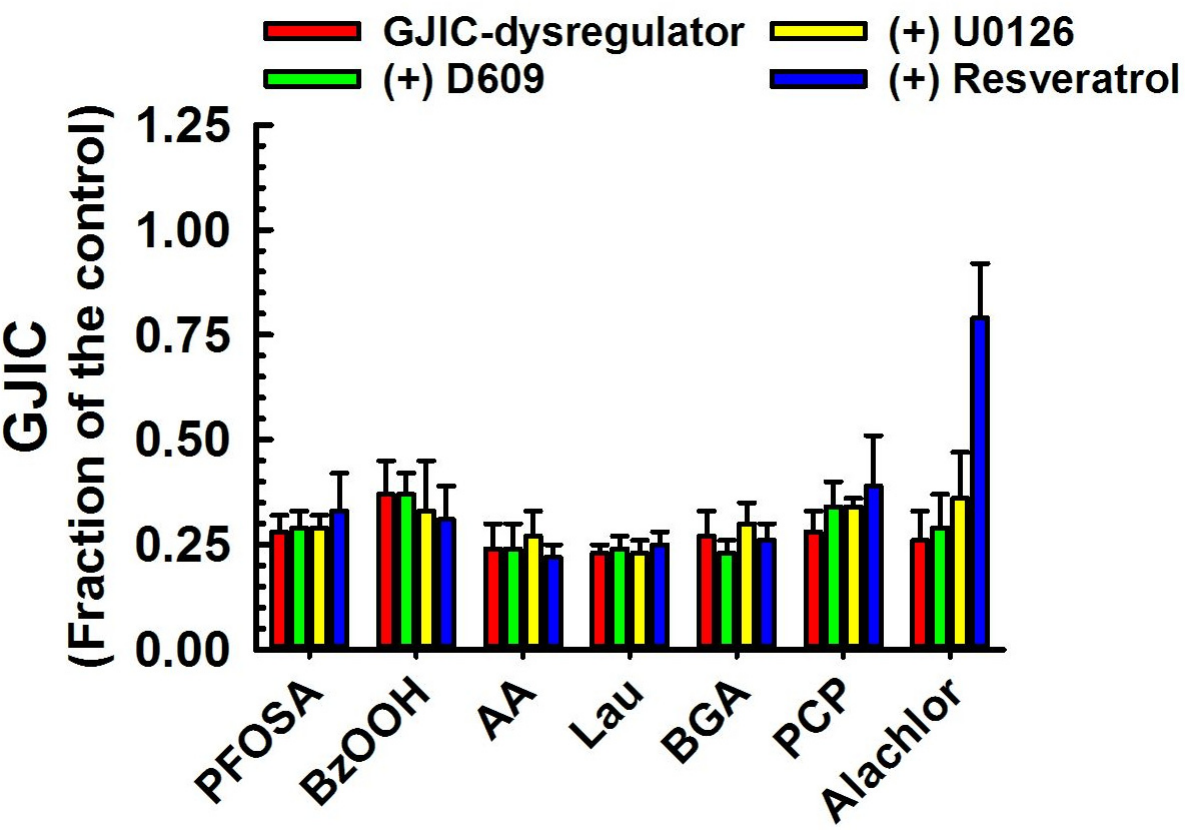
Dysregulation of GJIC through signaling pathways other than MEK1/2 or PC-PLC. The following compounds inhibited GJIC neither through MEK1/2 nor PC-PLC: PFOSA (40 μM, 20 min), BzOOH (200 μM, 15 min), AA (70–100 μM, 15 min), Lau (150 μM, 10 min), BGA (30 μμM, 15 min), PCP (50 μM, 10 min) and Alachlor (185 μM, 25 min). The cells were treated with inhibitors of PC-PLC (D609, 50 μM, 20 min) or MEK1/2 (U0126, 20 μM, 30 min), or resveratrol (100 μM, 15 min) before addition of GJIC-dysregulator. At least three independent experiments were averaged ± SD. An ANOVA was conducted for each GJIC-dysregulator followed by a Dunnett’s post-hoc test to determine significance (at P<0.05 as indicated by an *) from cells treated with only the GJIC-dysregulator. The F-values for PFOSA, BzOOH, AA, Lau, BGA, PCP and alachlor were 1.0 (P = 0.426), 0.6 (P = 0.628), 0.7 (P = 0.565), 0.6 (P = 0.617), 2.1 (P = 0.131), 1.9 (P = 0.162) and 58.6 (P<0.001), respectively.
